# Psychometric properties of the SDM-Q-9 questionnaire for shared decision-making in multiple sclerosis: item response theory modelling and confirmatory factor analysis

**DOI:** 10.1186/s12955-017-0656-2

**Published:** 2017-04-22

**Authors:** Javier Ballesteros, Ester Moral, Luis Brieva, Elena Ruiz-Beato, Daniel Prefasi, Jorge Maurino

**Affiliations:** 1 Department of Neurosciences and CIBERSAM, University of Basque Country, Leioa, Spain; 2 Department of Neurology, Hospital Moisés Broggi, Sant Joan Despí, Barcelona Spain; 3 Department of Neurology, Hospital Arnau de Vilanova, Lleida, Spain; 4 Health Economics and Outcomes Research Unit, Roche Farma SA, Madrid, Spain; 5 Medical Department, Roche Farma SA, Eucalipto 33, 28016 Madrid, Spain

**Keywords:** SDM-Q-9, Shared decision-making, Multiple sclerosis, Psychometrics, Patient involvement

## Abstract

**Background:**

Shared decision-making is a cornerstone of patient-centred care. The 9-item Shared Decision-Making Questionnaire (SDM-Q-9) is a brief self-assessment tool for measuring patients’ perceived level of involvement in decision-making related to their own treatment and care. Information related to the psychometric properties of the SDM-Q-9 for multiple sclerosis (MS) patients is limited. The objective of this study was to assess the performance of the items composing the SDM-Q-9 and its dimensional structure in patients with relapsing-remitting MS.

**Methods:**

A non-interventional, cross-sectional study in adult patients with relapsing-remitting MS was conducted in 17 MS units throughout Spain. A nonparametric item response theory (IRT) analysis was used to assess the latent construct and dimensional structure underlying the observed responses. A parametric IRT model, General Partial Credit Model, was fitted to obtain estimates of the relationship between the latent construct and item characteristics. The unidimensionality of the SDM-Q-9 instrument was assessed by confirmatory factor analysis.

**Results:**

A total of 221 patients were studied (mean age = 42.1 ± 9.9 years, 68.3% female). Median Expanded Disability Status Scale score was 2.5 ± 1.5. Most patients reported taking part in each step of the decision-making process. Internal reliability of the instrument was high (Cronbach’s α = 0.91) and the overall scale scalability score was 0.57, indicative of a strong scale. All items, except for the item 1, showed scalability indices higher than 0.30. Four items (items 6 through to 9) conveyed more than half of the SDM-Q-9 overall information (67.3%). The SDM-Q-9 was a good fit for a unidimensional latent structure (comparative fit index = 0.98, root-mean-square error of approximation = 0.07). All freely estimated parameters were statistically significant (*P* < 0.001). All items presented standardized parameter estimates with salient loadings (>0.40) with the exception of item 1 which presented the lowest loading (0.26). Items 6 through to 8 were the most relevant items for shared decision-making.

**Conclusions:**

The SDM-Q-9 presents appropriate psychometric properties and is therefore useful for assessing different aspects of shared decision-making in patients with multiple sclerosis.

## Background

The shared decision-making (SDM) is rooted in the ethical concept of bidirectional communication between patients and clinicians to reach agreements related to therapeutic issues. The basis for SDM is the principle of respecting individuals’ autonomy and their involvement in the discussion of available therapeutic options [1, 2]. Furthermore, SDM incorporates both patient values and preferences as well as the best available evidence related to treatment efficacy and safety. The goal of SDM is to improve the quality of patient care, health outcomes, and treatment adherence. A direct relationship between improved SDM quality and enhanced satisfaction with health care decisions has been previously documented [3].

Treatment decisions in multiple sclerosis (MS) are becoming more challenging due to increasingly diverse risk-benefit spectrum associated with the new disease-modifying therapies (DMTs) and the lack of reliable treatment response biomarkers [4, 5]. A more proactive management strategy, including earlier use of high-efficacy DMTs and close monitoring of the clinical and radiological response to treatment, is recommended to slow the progression of physical and cognitive impairments in patients with relapsing-remitting MS (RRMS) [6]. In this context, SDM may be a valuable tool in the clinical care of MS [7].

There are several instruments available to assess SDM in a clinical setting [8]. One of the most frequently used instruments is the 9-item Shared Decision-Making Questionnaire (SDM-Q-9), that has been translated and validated in Spanish amongst other languages [9, 10]. However, little is known about its psychometric properties in MS patients who must decide among alternative therapeutic options. Accordingly, and to generalize its application, the SDM-Q-9 was assessed in a population of patients with relapsing-remitting multiple sclerosis (RRMS). The aims of this study were to assess the psychometric performance of the items composing the SDM-Q-9 by item response theory (IRT) analysis, and to assess the unidimensionality of the instrument by confirmatory factor analysis (CFA).

## Methods

A non-interventional, cross-sectional study to assess patients’ preferences for a range of disease-modifying therapy attributes was conducted in 17 MS units throughout Spain (the EMPOWER study) [11]. Patients with the following inclusion criteria were enrolled in the study: (i) age ≥ 18 years, (ii) a diagnosis of RRMS according to McDonald 2010 criteria, (iii) currently receiving a DMT (at least during the previous three months), and (iv) an Expanded Disability Status Scale (EDSS) score of 1 to 6 points [12, 13]. Written informed consent was obtained from all subjects. The study was approved by the institutional review board of the Hospital Universitari Dr. Josep Trueta (Girona, Spain) and conducted between January and March 2016.

We performed a post hoc analysis using data from the aforementioned study in order to assess the psychometric performance of the SDM-Q-9. The SDM-Q-9 instrument was developed to assess the patient subjective experience of SDM according to nine stages on the decision-making process [9]. It describes the experience of SDM through nine items which are scored on a six-point Likert scale from 0 (completely disagree) to 5 (completely agree). Summing up all items leads to a raw total score between 0 and 45. As suggested we performed a linear transformation of the scale to provide a transformed score range from 0 to 100, with higher values indicating greater extent of SDM. The SDM-Q-9 has been shown to have high internal consistency, validity, and a unidimensional structure for the underlying construct it claims to measure [9]. Its main use is in studies investigating the effectiveness of interventions aimed at the implementation of SDM and as a quality indicator in health services assessments. We used the Spanish validated version of the SDM-Q-9 instrument [10].

### Statistical analyses

For continuous data, descriptive statistics were expressed as mean and standard deviation (SD), or median and interquartile range (IQR). For categorical data, descriptive statistics were expressed as frequencies and percentages. For description and comparison with other studies, and to aid the interpretation of the scoring, SDM-Q-9 scores were linearly transformed from the numerical value (0 to 5) to a percentage of the maximum score (0 = no SDM behaviour; 100 = ideal SDM behaviour) as suggested by the original authors of the scale [9]. However, for IRT and CFA the covariance matrix of raw scores were analyzed (range of 1 to 6).

A nonparametric IRT analysis (Mokken analysis) was used to assess the latent construct and dimensional structure underlying the association matrix of observed responses [14, 15]. Two Mokken models were fitted: the monotone homogeneity model and the double monotonicity model. The monotone homogeneity model tested the validity of a scale total score for ordering and classifying subjects according to the degree of the construct exhibited. The double monotonicity model was a more restrictive model in that it identified whether an order existed among the items and rated the corresponding construct that was independent of the selected sample. The Mokken results were interpreted according to the following rules: to be considered relevant, all items were required to have a scalability coefficient (*H*_*i*_) ≥ 0.30, and the total scale was required to have a scalability coefficient (*H*) ≥ 0.30. Mokken suggested the following thresholds for interpreting scalability coefficients for a measurement scale: weak scale 0.3 ≤ *H* < 0.4; medium scale 0.4 ≤ *H* < 0.5; and strong scale *H* ≥ 0.5 [14]. The internal reliability of the SDM-Q-9 scale was evaluated according to both Cronbach and Mokken estimates.

A parametric IRT model, the General Partial Credit Model (GPCM) was fitted to obtain estimates of the relationship between the latent construct and the item characteristics [16]. Specifically, the item response characteristic curve parameters (ICC) and the item information were estimated to assess the discrimination among category thresholds, and the measurement precision of the trait of each item, respectively.

Finally, the dimensionality of the SDM-Q-9 was assessed by confirmatory factor analysis (CFA). Goodness-of-fit for the CFA was evaluated using the comparative fit index (CFI) that evaluates the fit of a user-specified solution in relation to a more restricted, nested baseline model (null model), and the root-mean-square error of approximation (RMSEA) that assesses the extent to which a model reasonably fits in the population. A value of CFI > 0.95 was considered as an acceptable model fit, a RMSEA value < 0.08 was considered to reflect an adequate fit to the model, and a RMSEA value < 0.05 was considered as a good fit [17].

The statistical program R v3.3.1 with the libraries “*mokken*”, “*ltm*”, and “*lavaan*” was used to perform the statistical analyses [18–21].

## Results

A total of 221 patients were included in the study. Patients were predominantly female (68.3%) with a mean age of 42.1 ± 9.9 years. The mean time elapsed since diagnosis was 9 years and the median EDSS score was 2.5 ± 1.5. The most common current DMTs were first-line injectable therapies (43.9% of patients), followed by fingolimod (19.0%), dimethyl fumarate (15.4%), and natalizumab (12.2%). The main socio-demographic and clinical characteristics of the sample are presented in Table 1.

**Table 1:** Main characteristics of the sample

		*N* = 221
Age, mean (SD)		42.1 (9.9)
Gender. Female, *n* (%)		151 (68.3%)
Employment status, *n* (%)	Employed (part-time or full-time)	115 (52.0%)
	Unemployed	28 (12.7%)
	Retired due to MS	41 (18.6%)
	Retired due to other reasons	6 (2.78%)
	Without paid employment	31 (14.0%)
Some level of incapacity for work, *n* (%)		75 (34.9%)
Time of MS evolution (years), mean (SD)		9.1 (6.9)
EDSS score, median (SD)		2.5 (1.5)
Time with DMT treatment (years), mean (SD)		6.0 (4.4)
Use of previous DMT treatment, *n* (%)		133 (60.2%)
Presence of relapses, *n* (%)	Since diagnosis	201 (91.0%)
	During the last 2 years	100 (45.2%)
	During the last year	52 (23.5%)

DMT: Disease-modifying treatment; EDSS: Expanded Disability Status Scale; MS: multiple sclerosis

Median overall SDM-Q-9 score among the patients was 93.3% (IQR = 80-100%). The maximum SDM-Q-9 score was given by 62 (28%) of the patients. All SDM-Q-9 items scored high with maximum scores ranging from 54.3% (item 8:*”My doctor and I selected a treatment option together”*) to 77.1% (item 3:*”My doctor told me that there are different options for treating my medical condition”*).

### Internal reliability

The Cronbach’s α reliability coefficient was 0.91 (95% confidence boundaries = 0.89 to 0.93), and the Mokken reliability was 0.92.

### Non-parametric (Mokken) IRT

Table 2 shows the endorsement frequencies and Mokken scalability indices for the SDM-Q-9 items. All items were well-fitting for the unidimensional scale with the exception of item 1 (*“My doctor made clear that a decision needs to be made”*). Likewise, all items showed scalability indices (*H*_*i*_) over 0.30, except for item 1. The scalability for the overall scale (*H*) was 0.57 indicative of a strong scale according to Mokken’s criteria. The SDM-Q-9 scale well-fitted the Mokken monotonicity model with different item discrimination (no assumption violations were found), but not to the double monotonicity model which showed a large number of assumption violations for all items.

**Table 2:** Endorsement frequencies and Mokken scalability (Loevinger’s *H*_*i*_ coefficients) for the 9 items of the Shared Decision-Making Questionnaire (SDM-Q-9)

Item number and content	Mean (SD)	Endorsement frequencies (%)						*H*_*i*_
		1	2	3	4	5	6	
1. *A decision needs to be made*	5.19 (1.37)	14 (6)	4 (2)	3 (0.01)	18 (0.08)	49 (0.22)	135 (0.60)	0.27
2. *How I want to be involved*	5.17 (1.23)	7 (3)	5 (2)	7 (0.03)	31 (0.14)	46 (0.21)	127 (0.57)	0.60
3. *Informing me different options are available*	5.53 (1.12)	8 (4)	2 (1)	4 (0.02)	8 (0.04)	29 (0.13)	172 (0.77)	0.58
4. *Explaining me advantages and disadvantages*	5.57 (1.00)	5 (2)	2 (1)	3 (0.01)	12 (0.05)	31 (0.14)	170 (0.76)	0.59
5. *Helping me to understand the information*	5.63 (0.86)	4 (2)	0 (0)	2 (0.01)	10 (0.04)	37 (0.17)	170 (0.76)	0.54
6. *Asking the treatment option I prefer*	5.07 (1.46)	12 (5)	10 (4)	10 (0.04)	19 (0.08)	40 (0.18)	132 (0.59)	0.63
7. *Weighing jointly the different options*	5.13 (1.37)	11 (5)	8 (4)	4 (0.02)	25 (0.11)	46 (0.21)	129 (0.58)	0.66
8. *Selecting a treatment option together*	5.04 (1.41)	13 (6)	7 (3)	6 (0.03)	28 (0.13)	48 (0.21)	121 (0.54)	0.66
9. *Agreeing on how to proceed*	5.30 (1.23)	7 (3)	8 (4)	5 (0.02)	10 (0.04)	53 (0.24)	140 (0.63)	0.61

The overall scalability (*H*) is 0.57. Endorsement frequencies: 1 completely disagree; 2 strongly disagree; 3 somewhat disagree; 4 somewhat agree; 5 strongly agree; 6 completely agree

### Item characteristics: GPCM

**Fig. 1:**
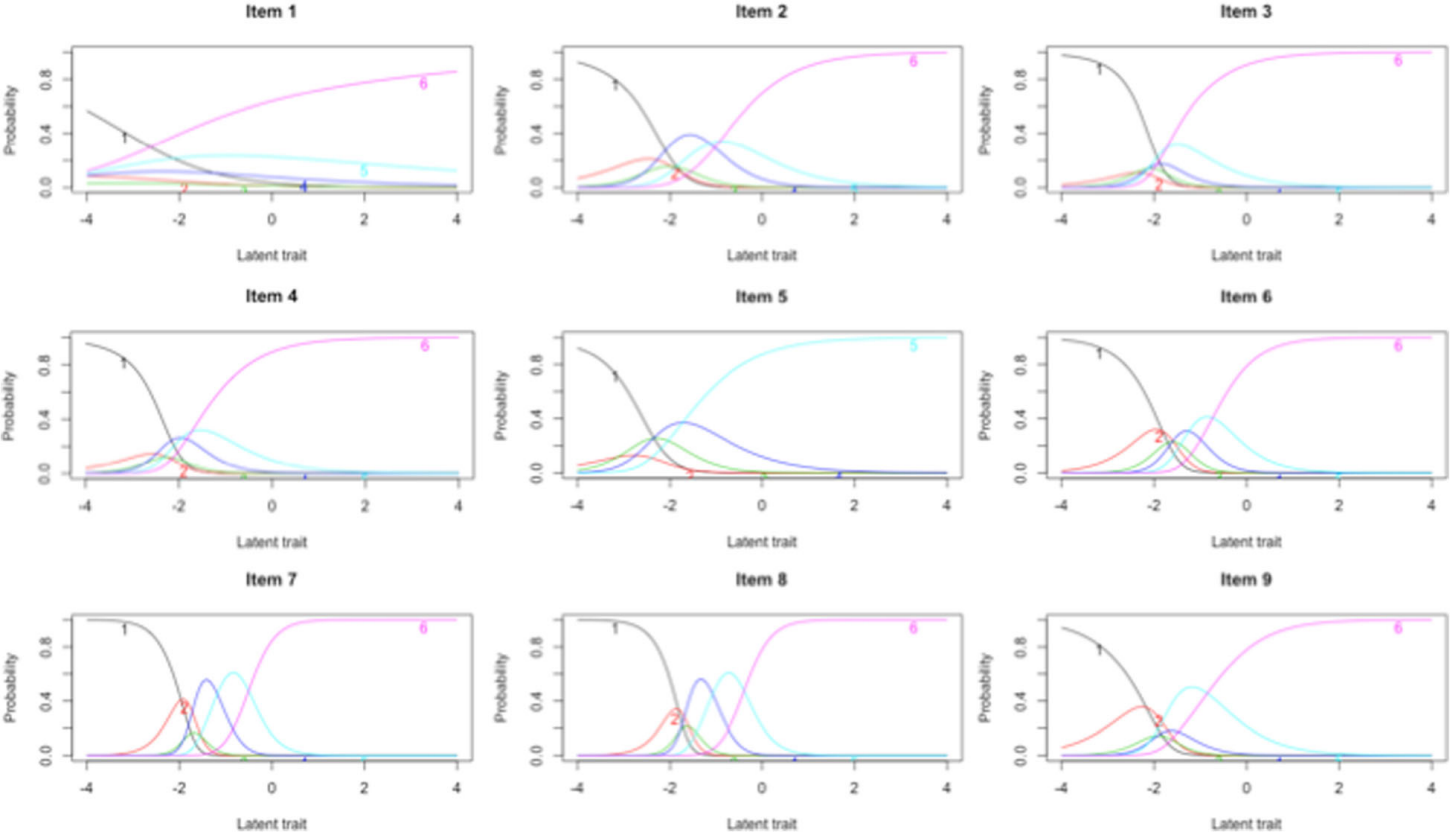
Item characteristic curves

Table 3 shows the parameters estimated by GPCM. More than half (67.3%) of the SDM-Q-9 overall information was conveyed by four items: item 7 (*“My doctor and I thoroughly weighed the different treatment options”*), item 8, item 6 (*“My doctor asked me which treatment option I prefer”*), and item 9 (*“My doctor and I reached an agreement on how to proceed”*). The least amount of information was conveyed by item 1 (1.5%). Figure 1 displays the ICC. Most items presented a shape and category threshold compatible with appropriate difficulty and discrimination parameters. As before, the main exception was item 1 which did not present a good behaviour.

**Table 3:** General Partial Credit Model (GPCM) parameters, and standardised loadings from confirmatory factor analysis for the 9-item Shared Decision-Making Questionnaire (SDM-Q-9)

Item no.	a	b_1_	b_2_	b_3_	b_4_	b_5_	Information	CFA loadings
1	0.22	4.50	0.42	−8.60	−4.83	−4.62	1.10	0.26
2	1.16	−1.73	−1.97	−2.59	−1.14	−0.93	5.80	0.72
3	1.34	−1.09	−2.40	−2.12	−2.14	−1.73	6.71	0.75
4	1.28	−1.51	−2.25	−2.72	−1.93	−1.73	6.40	0.67
5	1.15	−1.57	NA	−3.13	−2.36	−1.73	4.62	0.55
6	1.90	−1.83	−1.59	−1.61	−1.25	−0.74	9.48	0.87
7	3.41	−1.97	−1.53	−1.92	−1.18	−0.50	17.03	0.92
8	3.33	−1.83	−1.60	−1.79	−1.08	−0.39	16.66	0.92
9	1.52	−2.17	−1.48	−1.92	−2.11	−0.85	7.62	0.76

Goodness-of-fit indices for the CFA; CFI = 0.984, RMSEA = 0.069

Note a: discrimination parameter; b’s: threshold parameters; CFA: confirmatory factor analysis

### Factor structure

The SDM-Q-9 results well-fitted a unidimensional latent structure (CFI = 0.98, RMSEA = 0.07). Inspection of standardized residuals and modification indexes indicated no localized points of ill fit in the final solution with the exception of including correlated measurement error between some items (items 1 and 2; item 3 with items 4, 8 and 9; item 4 and 5; and item 5 and 9). All freely estimated unstandardized parameters were statistically significant (*p* < 0.001). Table 3 shows the standardized parameter estimates (factor loadings) for all items, all of which had salient loadings (>0.40), except for item 1 which had a salient loading of 0.26 and presented the lowest loading of all items. Items 6, 7 and 8 were the more related within the construct of SDM.

## Discussion

This study reports the psychometric properties of the SDM-Q-9 in a sample of patients with RRMS. Our results show that the SDM-Q-9 instrument is a worthwhile and feasible tool to elicit SDM in patients receiving treatment for MS. With the exception of item 1, the rest of the items of the scale fit to a unidimensional and strong structure according to Mokken criteria. The unidimensional structure of the instrument was also confirmed by CFA where all items (except for item 1) presented salient loadings (>0.40) within the construct of SDM. Furthermore, the internal reliability of the scale was high (Cronbach’s α = 0.91) and consistent with the range 0.88 to 0.94 found in other studies [9, 10, 22, 23].

Our results confirm previous observations of the SDM-Q-9 instrument which indicated that item 1 (*“recognizing that a decision needs to be maden*) did not present good psychometric properties and behaved differently from the rest of the items [9, 10, 22]. Item 1 had: (i) the lowest Mokken scalability index (*H*_*i*_ = 0.27) in an otherwise strong scale (*H* = 0.57); (ii) the lowest standardized CFA loading (0.26) - far from the usual criterion of salient loading with the underlying construct (values ≥ 0.40) which is achieved by the rest of the SDM-Q-9 items –; and (iii) the lowest discrimination parameter and information according to the IRT analysis. In fact, other studies have demonstrated that the fitting of the CFA analyses to a unidimensional structure improved when item 1 was excluded from the solution [6, 24]. It is likely that by modifying the content of the wording of the item, its ICC shape and psychometric properties would improve. However, we are not aware of any improvement of this scale through content modification, probably because of the high internal reliability attained by the current version of the SDM-Q-9 with relatively few items.

Our study has several limitations. First, the study population was comprised of a sample of clinically stable patients with low disability, mostly employed and receiving their first disease-modifying therapy. The results may thus not be generalizable to less stable subjects. Second, it would have been interesting to assess the absence of differential item functioning (DIF) in order to confirm the unidimensionality of the SDM-Q-9. However, there is not enough sample size to conduct a DIF appropriately according to classification criteria of the EMPOWER study. Despite this limitation, the study also has several strengths. The sample of 221 patients was managed in 17 different MS units at national level, which allows results to be generalised to community practice.

Shared decision-making is a cornerstone of patient-centred care [25]. Involving MS patients in the decision-making process is crucial for selecting the treatment that best suits the patient’s profile and preferences [7]. Therefore, treatment decisions in MS should be conjointly made by the neurologist and the patient, and should be based not only on the best available evidence but also taking into account the patient’s values and preferences [26, 27].

## Conclusion

The SDM-Q-9 instrument showed good psychometric properties and fitted a unidimensional latent trait related with SDM in patients with RRMS. Further studies focusing on the psychometric properties of the SDM-Q-9 instrument should be performed on representative samples of patients with other diseases benefitting from SDM processes in order to evaluate its equivalence.
